# Modulation of Microbiome–Mitochondria Axis as a Novel Approach for Treatment of Obesity: A Scoping Review

**DOI:** 10.3390/medsci14010124

**Published:** 2026-03-06

**Authors:** Andreea Roxana Lista, Ciskey Vanessa Ayala Mosqueda, Rafael Palacios, María José García Mansilla, María Jesús Rodríguez Sojo, Ailec Ho Plágaro, Jorge Garcia Garcia, Julio Gálvez, Alba Rodríguez Nogales, Antonio Jesús Ruiz Malagón, María José Rodríguez Sánchez

**Affiliations:** 1 Instituto de Investigación Biosanitaria de Granada (ibs.GRANADA), 18012 Granada, Spain; andreea99lista@gmail.com (A.R.L.); ciskey@correo.ugr.es (C.V.A.M.); centellito@correo.ugr.es (R.P.); mjgarcia@fibao.es (M.J.G.M.); ailec_hp@hotmail.com (A.H.P.); jgalvez@ugr.es (J.G.); albarnogales@gmail.com (A.R.N.); a.jesus.ruiz14@gmail.com (A.J.R.M.); mjrs2188@gmail.com (M.J.R.S.); 2 Department of Pharmacology, Centro de investigación Biomédica (CIBM), University of Granada, 18071 Granada, Spain; 3 CIBER de Enfermedades Hepáticas y Digestivas (CIBER-EHD), Instituto de Salud Carlos III, 28029 Madrid, Spain

**Keywords:** mitochondria, obesity, microbiota, microbiome-mitochondria axis, treatment

## Abstract

**Background:** Obesity is a multifactorial, chronic disease characterised by excessive fat accumulation, low-grade inflammation, and metabolic dysfunction. Emerging evidence suggests that the gut microbiome–mitochondria axis may play a significant role in the pathophysiology of obesity, particularly in regulating energy metabolism, inflammatory responses, and mitochondrial function. However, most mechanistic insights into this axis derive from preclinical animal studies, while human evidence remains limited and largely associative. Mitochondrial dysfunction disrupts cellular energy balance, increases reactive oxygen species production, and may exacerbate gut dysbiosis, further contributing to metabolic disturbances. In addition, factors such as micronutrient deficiencies also play a relevant role in obesity development and progression. **Objectives**: This review aims to examine the bidirectional interactions between the gut microbiome and mitochondrial systems in obesity, with a focus on the underlying molecular mechanisms and their potential as therapeutic targets. **Methods**: Evidence from experimental models and clinical studies was analysed to evaluate how modulation of the microbiome–mitochondria axis through probiotics, prebiotics, dietary strategies, and faecal microbiota transplantation influences mitochondrial function, inflammation, and metabolic regulation. **Results**: Preclinical studies indicate that the gut microbiome modulates mitochondrial activity through the production of bioactive metabolites, including short-chain fatty acids, secondary bile acids, and tryptophan-derived compounds, which influence mitochondrial efficiency, lipid metabolism, and glucose regulation. Dysbiosis reduces these beneficial metabolites, impairing mitochondrial signalling and promoting adiposity and insulin resistance. Interventions targeting this axis have shown potential in restoring metabolic balance, improving mitochondrial function, and mitigating obesity-related complications such as hyperlipidaemia and glucose intolerance. **Conclusions**: Targeting the microbiome–mitochondria axis represents a promising therapeutic strategy for obesity, with the evidence based largely on preclinical findings. However, further well-designed human studies are required to clarify causality, optimise interventions, assess long-term safety and efficacy, and establish standardised clinical protocols for implementation.

## 1. Introduction

Obesity is a chronic multifactorial disease characterized by excessive adiposity and metabolic dysfunction, which current affects approximately 16% of the global population [1]. Beyond excess weight, obesity is associated with chronic low-grade inflammation, oxidative stress, and systemic metabolic disturbances that contribute to cardiometabolic complications [2,3]. Therefore, obesity is a risk factor for metabolic syndrome, type 2 diabetes, hypertension, cardiovascular diseases, and several cancers [4,5].

Mitochondria are dynamic organelles involved in energy production, primarily through ATP synthesis. They also play crucial roles in apoptosis, calcium homeostasis, and protein and metabolite synthesis. Due to their evolutionary link to prokaryotes, as per the endosymbiotic theory, they share similarities with the gut microbiome [6,7].

Thus, the gut microbiome has shown to influence mitochondrial activity through the production of metabolites such as short-chain fatty acids (SCFAs), mainly butyrate, acetate and propionate. In addition, oxidative stress, induced by excess reactive oxygen species (ROS) produced by mitochondria, further alters the composition of the gut microbiome, thereby compromising the function of the intestinal barrier. Consequently, intricate networks of interactions between mitochondria and microbiome define the gut–brain axis and regulate inflammatory status, energy metabolism and gut homeostasis [7,8,9].

**Figure 1:**
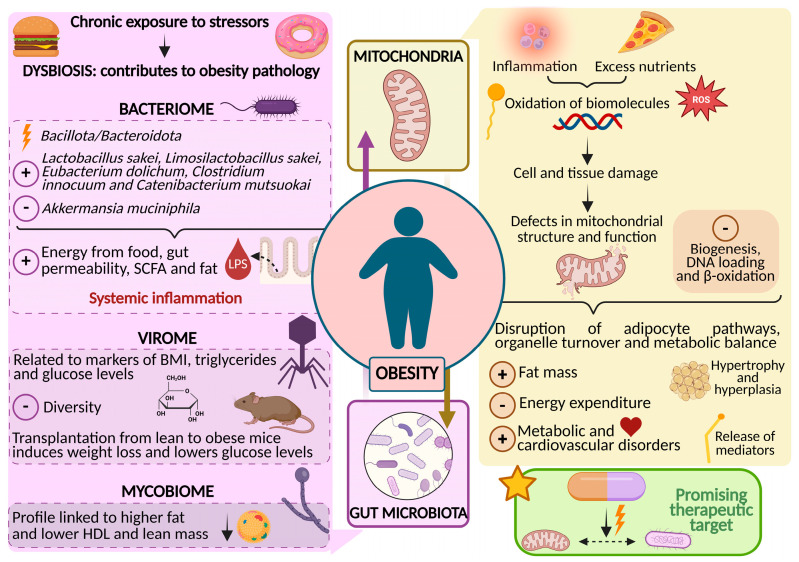
Schematic representation of the microbiota–mitochondria axis in the pathophysiology of obesity.

Despite the pathophysiology of obesity has not been fully elucidated, it is known that several mechanisms regulated by the bidirectional interaction between gut microbiome and mitochondria are altered in individuals with obesity [8]. Specifically, it is known that people with obesity have compromised mitochondrial metabolism [10]. Moreover, the gut microbiome also appears to play a key role in the onset and progression of obesity and related diseases, such as T2D and insulin resistance, by modulating appetite and influencing adipose tissue metabolism through its interaction with mitochondria [8,9] (Figure 1).

The diagram illustrates key mitochondrial alterations (impaired bioenergetics, oxidative stress, defective biogenesis and dynamics, and mitochondrial DNA damage) together with shifts in the gut microbiota, including changes in the bacteriome, virome, and mycobiome. It also depicts the bidirectional interactions between these systems and their modulation by diet. These interconnected alterations contribute to inflammation, metabolic dysfunction, and adipose tissue expansion, highlighting the microbiota–mitochondria axis as a relevant target for future therapeutic strategies in obesity.

Despite the magnitude of the problem and the associated healthcare costs, the long-term management of obesity remains challenging. Historically, pharmacological and lifestyle-based interventions have often produced modest and difficult-to-maintain weight reductions. More recently, the introduction of incretin-based therapies (e.g., GLP-1 receptor agonists and dual agonists) has substantially improved clinical outcomes and enabled clinically meaningful, sustained weight loss in many patients. However, important limitations persist, including poor long-term adherence, cost, variability in responses, and weight regain after discontinuation [11,12]. Moreover, key knowledge gaps remain regarding the extent to which these agents modulate mitochondrial function and the gut microbiome—two systems increasingly implicated in obesity pathophysiology. Emerging evidence suggests that incretin-based therapies may influence gut microbial composition and cellular energy metabolism, but mechanistic and longitudinal human studies are still limited.

In this context, the present scoping review aims to analyse the connection between the gut microbiome and mitochondria, with particular emphasis on the underlying molecular mechanisms and their potential as therapeutic targets. Additionally, it examines current and future strategies to modulate this axis through postbiotics, probiotics, prebiotics, and other innovative approaches, with the goal of contributing to the development of more effective and personalised long-term management strategies for obesity and its comorbidities.

## 2. Materials and Methods

To enhance transparency and reproducibility, this scoping review was conducted in accordance with the PRISMA-ScR (Preferred Reporting Items for Systematic Reviews and Meta-Analyses extension for Scoping Reviews) guidelines. A scoping review methodology was selected to systematically map the breadth of available evidence, identify knowledge gaps, and provide a comprehensive overview of the literature rather than a detailed evidence synthesis. The review protocol was developed a priori and subsequently reviewed and refined by the research team and collaborators. Although this scoping review was not prospectively registered in a public database, the methodology adhered to established methodological guidance for scoping reviews.

### 2.1. Eligibility Criteria

The inclusion criteria for this scoping review aimed to identify the most relevant and up-to-date studies on the subject. Eligible studies included preclinical research using animal models of obesity, clinical trials, and randomized controlled trials investigating the bidirectional relationship between the microbiome and mitochondrial function and dysfunction, as well as their implication in the development and treatment of obesity. Additionally, due to the limited number of experimental studies, some review articles were included to complement the findings from more recent studies. Therefore, the considered studies provide valuable insights into potential innovative agents or strategies that could target these interactions and improve the management of obesity.

To refine the scope of the review, specific exclusion criteria were applied. Studies without experimental validation, including opinion articles, editorial materials, and theoretical models lacking supporting data, were excluded. Furthermore, research that did not assess key outcomes related to the microbiome-mitochondria axis was omitted to ensure relevance to the study objective. This approach also excluded studies that focused solely on the isolated effects of microbiome modulation, as well as those that addressed the microbiome-mitochondria axis without employing experimental studies in models of obesity or related diseases, or clinical studies involving patients with these conditions.

### 2.2. Information Sources and SEARCH Strategy

Accordingly, a comprehensive and systematic search was conducted across major scientific databases, including PubMed, Scopus, and ScienceDirect. In addition, ResearchGate was consulted to identify potentially relevant publications, preprints, and grey literature that may not be consistently indexed in traditional bibliographic databases, thereby supporting the overall comprehensiveness of the scoping review. All records identified through ResearchGate were subsequently screened according to the predefined eligibility criteria.

The computerized search was restricted to scientific articles published between 2015 and 2025, with the final search conducted on 10 September 2025. During this process, the strategy used was based on the use of Boolean operators and combinations of key terms highlighting “obesity”, “mitochondrial dysfunction”, “gut microbiome”, “microbiome-mitochondria axis”, “microbiota”, “microbiome”, “mitochondrial-targeted treatments”, “probiotics”, “prebiotics”, “postbiotics” and “signalling pathways”. When studies were duplicated and/or overlapping in different databases, only one of them was selected.

### 2.3. Data Quality Assessment, Extraction, and Grouping

Study selection and data extraction were conducted independently by two reviewers. Any discrepancies were addressed through discussion, and when a consensus could not be reached, a third reviewer was consulted to make the final decision. Although inter-rater reliability was not formally quantified (e.g., Cohen’s kappa), all disagreements were documented and resolved through consensus before the analysis. For data extraction, a checklist was created considering the selection criteria. This list included details such as publication year, authors, type of study (preclinical or clinical), participants, sample size, reported changes in the microbiota, among others.

## 3. Results and Discussion

**Figure 2:**
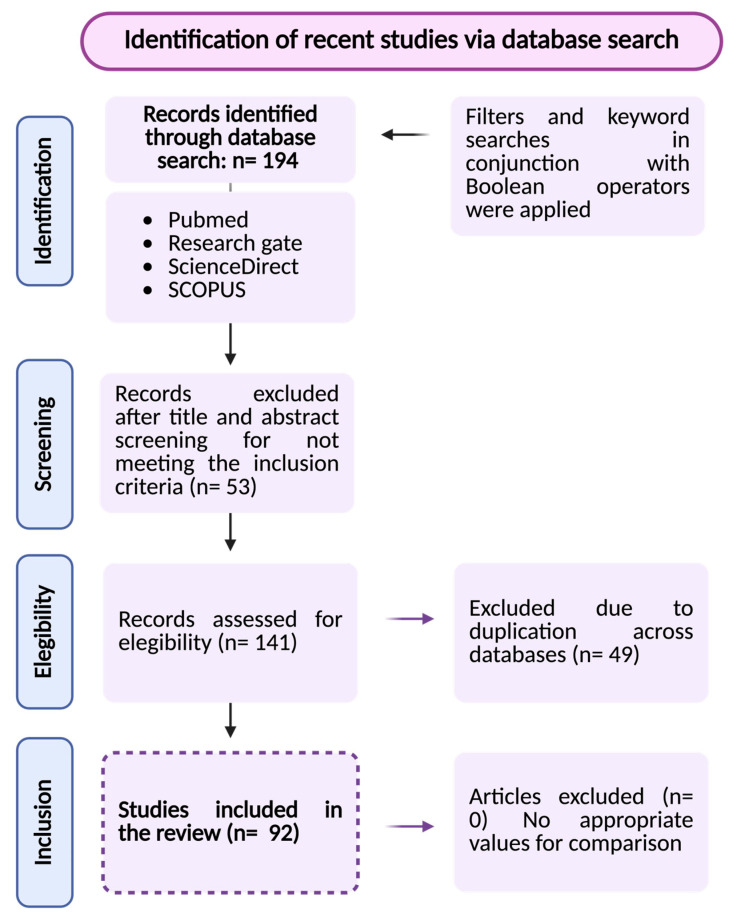
PRISMA flow diagram illustrating the review process, including database searches, application of inclusion and exclusion criteria, and final selection of studies, in accordance with PRISMA guidelines.

A total of 194 entries were initially identified through the database searches considering the predefined inclusion and exclusion criteria. Then, 53 records were excluded after title and abstract screening and 141 studies were included in the final analysis, encompassing both preclinical and clinical research. Subsequently, 49 duplicates were excluded. After thoroughly reviewing the titles and abstracts of the remaining articles, 92 were deemed potentially relevant for inclusion in this review (Figure 2).

These included studies offer valuable insights into the bidirectional interactions between the mitochondrial organelle and the gut microbiome, particularly in the context of obesity development and therapeutic interventions. Furthermore, this review emphasizes specific agents with the potential to influence disease progression by modulating these complex interactions. By focusing on evidence-based mechanisms and clinical applicability, the analysis highlights promising strategies within the framework of obesity research.

### 3.1. Gut Microbiome: A Key Player in Triggering Obesity

The human gut microbiome—a dynamic and intricate community of microorganisms including bacteria, viruses, fungi, archaea, and protozoa—has forged a deeply symbiotic relationship with its host [13]. This “invisible” ecosystem performs a wide array of vital functions that support gut physiology and overall health: regulating epithelial cell proliferation and differentiation [14], influencing insulin resistance and secretion [13], shaping brain–gut communication and thus neurological and psychological health [14], synthesizing vitamins, warding off pathogens, fermenting fiber, extracting energy, and modulating immune development [15,16,17].

Interestingly, the microbial landscape differs across sections of the gastrointestinal tract. The oesophagus and stomach harbour fewer microbes due to their acidic and oxidative environments, while the microbial population explodes in density and diversity further down the tract. The small intestine is relatively sparse and dominated by *Proteobacteria* and *Clostridium*, whereas the colon is one of the densest microbial habitats on Earth (10^11^–10^12^ bacterial cells/mL), teeming primarily with *Clostridium* and *Bacteroidota* [18].

Most studies focus on the bacterial component—known as the bacteriome. Despite variability across populations, age, and sex [19,20], the dominant phyla in the human gut are *Bacillota* (formerly *Firmicutes*), *Bacteroidota*, *Proteobacteria*, *Verrucomicrobia*, *Actinobacteria*, *Fusobacterium*, and *Cyanobacteria* [16,21]. While the microbiome demonstrates resilience to short-term changes, chronic exposure to stressors—such as a high-fat, Westernized diet—can cause dysbiosis or long-term disruption of microbial balance. This has profound consequences for host health, including a heightened risk for obesity [16,22]. Though genetics, lifestyle, and diet are established risk factors, research increasingly points to the gut microbiome as a critical contributor to obesity pathogenesis. In line with this, high-fat diets in mice have been shown to reduce populations of *Bacteroidota*, *Bacteroidales*, *Verrucomicrobiales*, and *Lactobacillus*, while increasing *Bacillota*, *Erysipelotrichales*, *Clostridiales*, and *Lachnospirales* [23].

In humans, individuals with obesity frequently display an altered *Bacillota* to *Bacteroidota* ratio, along with the increased presence of species such as *Lactobacillus sakei*, *Limosillactobacillus reuteri*, *Eubacterium dolichum*, *Clostridium innocuum*, and *Catenibacterium mitsuokai* [24,25,26]. These changes are linked to increased energy extraction from food and increased intestinal permeability, allowing endotoxins like lipopolysaccharides (LPSs) to enter circulation, triggering systemic low-grade inflammation—a hallmark of obesity [27,28]. Furthermore, individuals with obesity typically exhibit reduced levels of *Akkermansia muciniphila*, a mucin-degrading bacterium that helps maintain gut barrier integrity [29]. Altered microbiota profiles also lead to increased production of SCFAs, which may contribute to lipogenesis and gluconeogenesis, fueling fat accumulation [30].

### 3.2. Beyond Bacteria: The Role of the Virome and Mycobiome in Obesity

Thanks to advances in next-generation sequencing and bioinformatics, scientists are now uncovering the impact of non-bacterial microbes—notably viruses and fungi—on metabolic health [31,32].

#### 3.2.1. The Gut Virome and Its Emerging Role in Obesity

The virome consists of viruses that infect both microbial residents (bacteriophages or phages) and human cells, as well as transient dietary viruses. The gut is a hotspot for viral diversity, dominated by phages such as those from the *Caudovirales* order (*Myoviridae*, *Podoviridae*, *Siphoviridae*) and *Microviridae* [33,34]. Eukaryotic RNA viruses, including non-pathogenic plant viruses like *Picobirnaviridae* and *Virgaviridae*, are also common [35].

Factors influencing virome composition include diet, medication, aging, and chronic illness. Dysbiosis of the gut virome has been linked to a range of diseases including obesity, type 1 and 2 diabetes, irritable bowel disease, and metabolic syndrome [36,37,38]. In line with this, mice with obesity show higher viral DNA/RNA loads in feces, correlating with greater body weight, fat mass, and fasting glucose [39]. In humans, phages (especially *Caudovirales*) have been associated with obesity-related markers like BMI, triglycerides, and glucose levels [37]. Individuals with obesity—particularly those with T2D—tend to have lower gut viral diversity, with altered correlations between viruses and bacteria [31].

Promisingly, post-treatment changes in the virome have been observed: one study found that the number of core viruses increased from four (pre-treatment) to thirteen (post-treatment), along with higher diversity [40]. Even blood samples show links: higher seroprevalence of herpes simplex virus 1 has been positively associated with obesity, hinting at viral involvement in fat cell development [41]. Excitingly, studies in mice have shown that transferring the viral community from lean to obese animals led to reduced weight gain and improved blood sugar levels [42]. Together, these findings highlight the virome as a potential modulator of metabolic health, offering new avenues for understanding and possibly treating obesity.

#### 3.2.2. Fungal Footprints in Fat: The Mycobiome’s Role in Obesity

Fungi make up only a small fraction (0.03–2%) of the gut microbiome, but their impact is significant [43]. Using cutting-edge sequencing, researchers have catalogued over 66 genera and more than 278 fungal species in the human gut—primarily from *Ascomycota*, *Basidiomycota*, and *Zygomycota* [44,45]. Common genera include *Saccharomyces*, *Malassezia*, *Candida*, and *Cladosporium* [43,46]. Fungal profiles in obesity show distinct patterns. The abundance of the *Mucor* genus tends to drop, while *Candida albicans* is more prevalent in individuals with obesity [47]. These alterations also correlate with metabolic markers like fat mass, triglycerides, and HDL levels, and crucially, they may reverse with weight loss.

Additionally, recent investigations have demonstrated that, in patients with obesity, *Candida albicans*, *Candida kefyr*, and *Teunomyces krusei* display increased pathogenic potential than they do in individuals with normal weight [48]. This heightened virulence is associated with elevated expression of hydrolytic enzymes and an enhanced ability to form biofilms.

Although *Candida albicans* is commonly regarded as an opportunistic pathogen, recent evidence suggests it may also exert beneficial effects in specific contexts. *Candida albicans* has shown self-regulation of its abundance depending on dietary composition in preclinical studies [49]. In murine models fed a high-fat diet, it promoted healthy weight by attenuating dysbiosis and modulating key appetite-regulating hormones commonly altered in obesity, such as leptin, insulin, C-peptide, and resistin. Regulatory effects were also observed on GLP-1 and GIP. Through these mechanisms, *Candida albicans* reduced weight gain by 15%, decreased abdominal fat, improved lipid and glucose metabolism, and exerted a protective effect on renal function.

Strains such as *Candida kefyr* and *Rhodotorula mucilaginosa* show strong positive associations with obesity and fat mass, and negative associations with HDL and lean body mass [47]. These findings underscore the mycobiome’s dynamic role in metabolic health, positioning gut fungi as emerging players in the complex puzzle of obesity.

### 3.3. The Mitochondrial Dysfunction in Obesity

The pathogenic mechanisms associated with obesity have not been fully elucidated; however, it is well known to be a situation of chronic and subclinical systemic inflammation related to changes in cells of metabolic tissues in response to excess nutrients and energy [50]. Chronic overfeeding causes an enlargement of adipose tissue depots and an alteration in their functionality. Consequently, it increases the levels of free fatty acids and the production of inflammatory cytokines, which also contribute to the development of hypertrophy and hyperplasia of adipocytes. The excessive production of these mediators by adipose tissue and their release into the bloodstream has a relevant impact on other organs and systems, facilitating the appearance of metabolic and cardiovascular disorders such as diabetes or endothelial dysfunction that invariably accompany obesity [51].

On the other hand, it has also been described that the inflammation-related mechanisms that occur in obesity increase the production of ROS, thus resulting in oxidative stress that can lead to the oxidation of biomolecules, such as lipids, proteins and DNA [52,53]. Consequently, these biomolecules lose their biological functions and/or homeostatic imbalance, facilitating cell and tissue damage, and causing defects in mitochondrial structure and function as well [54]. As mentioned above, the primary function of the mitochondria is to produce energy in the form of ATP from food substrates. In addition, the mitochondrial respiratory chain generates ROS during ATP production and therefore represents the main source for the production of ROS [55]. In fact, it is well established that the excess nutrient intake associated with obesity can lead to mitochondrial ROS production and, in turn, cause mitochondrial dysfunction [54].

Obesity-associated mitochondrial dysfunction has been consistently reported in preclinical models and observed in human studies; however, most mechanistic insights derive from animal and in vitro research. This dysfunction occurs in multiple cell types, with adipose tissue, skeletal muscle, liver, and blood being the most affected. Alteration of mitochondrial function reduces mitochondrial biogenesis and its DNA load and diminishes the rate of β-oxidation [56,57]. Consequently, the adipocyte pathways, including adipogenesis, lipolysis, fatty acid esterification and adipocyte-derived adiponectin production are altered. In line with this, studies have revealed decreased mitochondrial biogenesis in adipose tissue, and a reduction in the mitochondrial oxidative capacity as well as its biogenesis in adipocytes from patients with obesity [58]. Similarly, defective mitochondrial function has also been reported in skeletal muscle in experimental studies of obesity in mice, which is related to a reduction in fatty acid oxidation and inhibition of glucose transport, which is indicative of insulin resistance [59]. Moreover, alteration of the hepatic mitochondria function in diet-induced obesity has been reported [60], which was characterized by reduction in mitochondrial protein expression, including expression of peroxisome proliferator-activated receptor-γ coactivator-1α (PGC-1α), a transcriptional coactivator that acts as central inducer of mitochondrial biogenesis in cells [61].

Additionally, mitochondrial alterations have also been reported to affect mitochondrial dynamics, including the coordinated cycles of fission and fusion that affect the shape, size and distribution of mitochondria. Consistent with this, altered levels of proteins involved in dynamic mitochondrial changes, such as mitochondrial fission 1 protein, dynamin-related protein 1, mitochondrial fusion protein mitofusin 1 and 2, and dominant optic atrophy, have been observed in both mice and humans with obesity [62]. Also, there are studies suggesting that obesity and its associated metabolic comorbidities can be related to defects in several processes of mitochondrial dynamics in peripheral blood leukocytes, which are widely known to be sensors of the responses to disease [63,64]. These mitochondrial alterations include changes to fusion/fission processes, the repair or removal of dysfunctional organelles and mechanisms of mitochondrial biogenesis, which lead to defects in metabolic homeostasis, increased adipose mass, and decreased energy expenditure that characterize obesity.

Most of the above-mentioned reports are from research in animal models, while evidence obtained in humans is scarce. Therefore, future investigations are necessary to define the essential role of mitochondrial dysfunction in determining obesity and, in turn, potentially opening new avenues for the treatment of this condition.

### 3.4. The Microbiome-Mitochondria Axis

The well-accepted endosymbiotic theory establishes that key organelles of the modern eukaryotic cell arose from the fusion and symbiotic relationship between proteobacteria organisms [65]. Additionally, it is interesting to note that mitochondria are inherited maternally, and the founding colonies of the microbiome in newborns are also from a maternal source [66]. Therefore, mitochondria and bacterial members of the microbiome share many features and origins. Indeed, degraded mitochondrial proteins or mitochondrial DNA can activate formylated protein receptors, similarly to that observed with microbial formylated proteins, to signal proteins in eukaryotic cells [67]. Both bacterial and mitochondrial membranes can be degraded through similar autophagic systems; mitochondrial and bacterial ribosomes are more related to each other than either is to eukaryotic ribosomes, and both are sensitive to antibiotics [68]. Moreover, it has been reported that diverse bacterial proteins can be imported into the host mitochondria due to the similarity of the mitochondrial bacterial cytoplasmic protein targeting sequence. Of note, mitochondria and bacteriome also share commonalities in terms of intercommunications, which have been suggested to control several metabolic pathways and/or processes, with SCFAs and secondary bile acids (SBA) being specifically used as key players in this communication [69].

Conversely, experimental data show a bidirectional communication between microbiome and mitochondria. It has been reported that alterations to mitochondrial functions can modify the gut microbiome composition and functionality because they are able to influence the activities of intestinal functional effector cells, such as immune and epithelial cells. Interestingly, polymorphisms of mitochondrial genes such as ND5 and CYTB genes or the D-Loop region in the mitochondrial genome have been associated with specific gut microbiome compositions [67,70,71,72]. Consequently, the contributing role of mitochondria on gut microbiome architecture is becoming evident.

Thus, given their crucial roles in regulating metabolic pathways, both gut microbiome and mitochondria have become central focuses in medical and biological research on obesity, potentially paving the way for new treatments for obesity.

### 3.5. Targeting the Microbiome–Mitochondria Connection: A New Frontier in Therapy

A substantial proportion of the mechanistic evidence supporting microbiome–mitochondria interactions derives from preclinical models. Although emerging human studies provide encouraging data, many of the reported effects should presently be interpreted as biologically plausible mechanisms rather than clinically established causal pathways. Accordingly, adequately powered and well-controlled clinical trials are required to confirm the translational relevance and therapeutic applicability of targeting the microbiome–mitochondria axis in metabolic diseases.

The conventional management of obesity primarily relies on dietary modification, increasing physical activity, and comprehensive lifestyle interventions. These strategies are frequently complemented by pharmacological therapies, including appetite suppressants, inhibitors of intestinal fat absorption, and, more recently, glucagon-like peptide-1 (GLP-1) receptor agonists such as liraglutide [73]. Despite their demonstrated efficacy—particularly for modern incretin-based agents—important real-world challenges remain, including poor long-term adherence, treatment discontinuation, economic burden, and heterogeneity in patient response. Notably, whether modern anti-obesity pharmacotherapies directly modulate the microbiome–mitochondria axis in humans remains largely unexplored and is supported by limited mechanistic evidence. Limited compliance, waning motivation, and difficulties in maintaining behavioral changes frequently lead to therapy discontinuation, thereby contributing to disease persistence and progression [74]. In light of the global prevalence of obesity and its wide range of associated comorbidities, the identification of alternative therapeutic strategies and novel molecular targets represents a critical unmet clinical need.

Within this framework, distinct therapeutic approaches have independently focused on correcting gut dysbiosis or ameliorating mitochondrial dysfunction. However, relatively few investigations have addressed these components in an integrated manner despite mounting evidence underscoring the functional relevance of the gut microbiota–mitochondria axis in obesity and related metabolic disorders. Although studies directly examining mitochondrial–microbiome interactions remain limited, available data consistently support the existence of a bidirectional crosstalk that significantly influences host metabolic homeostasis.

Experimental models of obesity have demonstrated that natural compounds, probiotics, prebiotics, and their derived metabolites can significantly reduce body weight, adiposity, and systemic inflammatory markers while simultaneously improving lipid profiles. These beneficial effects are closely associated with restoration of gut microbial balance and enhancement of intestinal barrier integrity [9].

Beyond their impact on gut homeostasis, biotics have also shown promising systemic effects, including improvements in metabolic parameters, blood pressure regulation, and cardiac function. These outcomes are largely attributed to their capacity to attenuate oxidative stress, suppress chronic inflammation, and mitigate mitochondrial dysfunction [75]. Notably, recent studies have identified specific mitochondrial targets modulated by these interventions, including uncoupling protein 1 (UCP-1), BCL-2-associated X protein (BAX), B-cell lymphoma 2 (BCL-2), and BCL-2 interacting mediator of cell death (BIM), all of which play central roles in mitochondrial dynamics, energy metabolism, and apoptosis regulation [76]. Collectively, these findings position the microbiome–mitochondria axis as a promising and mechanistically grounded therapeutic target, warranting further translational investigation in human populations.

#### 3.5.1. Prebiotics: Fuelling the Microbiome–Mitochondria Conversation

Recent research highlights how certain prebiotic compounds can mediate beneficial microbiome–mitochondrial interactions (Table 1). For instance, the co-administration of inulin and rhubarb extract has demonstrated the ability to reduce weight gain in mice through microbiome modulation. This intervention enhanced mitochondrial activity, reduced browning of brown adipose tissue, and elevated levels of lipolytic markers. It also influenced key transcription factors such as C/EBP and PPARγ, promoting an adaptive metabolic response. Moreover, rhubarb stimulated the production of SCFAs, further supporting their beneficial metabolic effects [77].

In a similar vein, type 3 resistant starch (Ce-RS3)—extracted from *Canna edulis*—has proven effective in modulating both metabolic outcomes and gut microbiota in preclinical obesity models. Ce-RS3 resists absorption in the small intestine, allowing it to serve as a substrate for fermentation in the colon. This fermentation promotes the growth of beneficial bacteria such as *Bifidobacterium* and *Roseburia*, reversing dysbiosis, reducing weight, dampening chronic inflammation, and improving lipid metabolism [78]. Interestingly, a randomized controlled trial by Miao et al. (2024) further supported these findings, showing that Ce-RS3 improved cholesterol levels and glutathione peroxidase activity in individuals with mild hyperlipidemia by modulating the phenylalanine metabolic pathway and enhancing populations of *Faecalibacterium* while reducing *Ruminococcaceae*, potentially limiting mitochondrial fatty acid synthesis [79].

Moreover, other resistant starches have shown comparable benefits. For instance, RS5 (type 5 resistant starch) was shown to improve dysbiosis, decrease body weight, and enhance lipid metabolism in mouse models of hyperlipidemia [80]. Furthermore, other preclinical studies using rat models fed high-fat diets also demonstrated that RS5 administration has the ability to reduce body weight and enhance lipid metabolism through the modulation of the microbiota [81]. Likewise, RT-90—a type 4 resistant starch derived from tapioca—demonstrated significant reductions in weight and markers of inflammation and oxidative stress. These effects were accompanied by increased activity of antioxidant enzymes such as GPx, SOD, and CAT, all tied to the restoration of SCFA-producing bacteria [82].

**Table 1:** This table summarizes the main findings reported for different prebiotics, including Inulin and rhubarb extract, Ce-RS3 (Resistant Starch Type 3 from Canna Edulis), RS5 (Type 5 Resistant Starch), and RT-90 (Resistant Starch Type 90).

Outcome	Prebiotic	Model
↓ Weight or weight gain	Inulin and rhubarb extract [77] Ce-RS3 [78] RS5 [80,81] RT-90 [82]	Murine
↓ Browning of brown adipose tissue	Inulin and rhubarb extract [77]	
↓ Dysbiosis	Inulin and rhubarb extract [77] Ce-RS3 [78] RS5 [81] RT-90 [82]	
↓ Inflammation	Ce-RS3 [78] RS5 [80] RT-90 [82]	
↓ Oxidative stress	RT-90 [82]	
↓ Adipocyte size	RS5 [80]	
↓ Glycemia		
↓ Fat absorption		
↓ Diabetes biomarker	RT-90 [82]	
	Ce-RS3 [78] RS5 [80]	
↓ Blood lipid levels	Ce-RS3 [79]	Individuals with mild hyperlipidaemia (human)
↓ Mitochondrial fatty acid synthesis		
↓ Mitochondrial elongation		
↑ Mitochondrial activity	Inulin and rhubarb extract [77]	Murine
↑ Lipid metabolism	RS5 [80,81]	
↑ Lipolytic markers	Inulin and rhubarb extract [77]	
↑ SCFAs	Inulin and rhubarb extract [77] RS5 [80,81] RT-90 [82]	
↑ Beneficial bacteria	Ce-RS3 [78]	
	Ce-RS3 [79]	Individuals with mild hyperlipidaemia (human)
↑ Microbial richness	RS5 [80]	Murine
↑ Antioxidant enzymes	RS5 [81] RT-90 [82]	

#### 3.5.2. Postbiotics: Microbial Metabolites That Rewire Mitochondrial Function

During the last years, postbiotics—particularly SCFAs like butyrate—have emerged as promising regulators of mitochondrial energy metabolism in preclinical models. Butyrate, which is produced primarily by *Bacillota*, has been shown to be able to stimulate mitochondrial biogenesis by inhibiting histone deacetylases, resulting in greater energy expenditure and body weight reduction [83].

In addition, bile acids (BAs) and SBAs produced by gut microbes play a role in regulating mitochondrial energy generation. They act through transcription factors that control lipid and carbohydrate metabolism and modulate NAD-dependent deacetylases, directly influencing mitochondrial functionality [84,85] (Table 2). A translational study combining clinical analysis of adults with elevated BMI—both metabolically healthy and unhealthy—and murine models prone or resistant to obesity identified a link between BA profiles and disease development [84]. Both metabolically healthy individuals and obesity-resistant mice showed a predominance of non-12-hydroxylated bile acids, such as ursodeoxycholic acid (UDCA), whereas unhealthy subjects and obesity-prone mice exhibited higher levels of 12-hydroxylated bile acids and an increased presence of bacteria like *Clostridium scindens*. These findings suggest a potential role of UDCA in modulating obesity-related metabolic pathways, as evidenced by reduced weight gain and increased expression of mitochondrial regulators such as PGC1α and UCP1 in brown adipose tissue, although confirmation in large-scale human studies is required.

#### 3.5.3. Probiotics Enhancing Mitochondrial Health

Probiotic strains such as *Bifidobacterium animalis* subsp. *lactis* A6, *Limosillactobacillus reuteri* DSM 17938 and *Akkermansia muciniphila* have shown anti-obesity-related metabolic improvements in preclinical models. These include reductions in body weight and adipose tissue mass, along with suppressed levels of pro-inflammatory LPS-producing bacteria like *Oscillibacter* and *Bilophila*. The probiotic treatments also enhanced the expression of mitochondrial regulators PGC-1α, ERRα, and UCP-1 in adipose tissues, suggesting a boost in mitochondrial biogenesis and function [75].

In this way, a study conducted in mice demonstrated that oral administration of pasteurized *Akkermansia muciniphila* effectively alleviated diet-induced obesity and reduced food energy efficiency. Along this study, Depommier and colleagues observed that this effect was accompanied by an increase in energy expenditure. Interestingly, they found that expression of perilipin2—a lipid droplet-associated protein typically upregulated in obesity—was reduced in both brown and white adipose tissues following treatment with pasteurized *Akkermansia muciniphila*. Furthermore, the treatment led to a notable increase in energy loss through feces that appeared to involve reduced carbohydrate absorption and enhanced turnover of the intestinal epithelium [86].

Recent research using rats fed with Western diet has shown that administering *Limosillactobacillus reuteri* DSM 17938 for eight weeks protects skeletal muscle and adipose tissue by preventing the onset of inflammation, insulin resistance, mitochondrial dysfunction, and oxidative stress. Notably, this probiotic also exerted a beneficial effect on body composition, promoting protein mass accumulation while limiting fat mass expansion. These findings lay the groundwork for the potential use of this probiotic in therapeutic strategies targeting nutrition-related conditions such as metabolic syndrome, obesity, and lean mass loss [87].

Regarding *Bifidobacterium*, a study conducted by Maruta aimed to evaluate the beneficial effects of milk fermented with different strains on energy metabolism and obesity prevention. The results showed that rats administered *Bifidobacterium longum*-fermented milk exhibited significantly lower weight gain compared to the water-treated control group. In the livers of these rats, AMPK phosphorylation was increased, while the expression of lipogenic genes was suppressed. Additionally, genes related to mitochondrial biogenesis and respiratory metabolism were significantly upregulated. These findings suggest that milk fermented with *Bifidobacterium longum* may enhance lipid metabolism and help prevent obesity [88].

Together, these findings suggest a potential therapeutic relevance that warrants further validation in controlled human trials. In fact, the modulation the gut microbiota with specific bacterial strains to influence systemic energy metabolism and combat metabolic disorders (Table 3).

#### 3.5.4. Natural Compounds: Targeting the Mitochondria–Microbiome Synergy

Among natural bioactive molecules (Table 4), rotundic acid—a pentacyclic triterpenoid—has demonstrated metabolically favorable effects, primarily in experimental settings in hyperlipidemic rats. It improved weight management, restored lipid homeostasis, and favored the growth of SCFA-producing gut bacteria [89]. Mechanistically, it modulated glycerophospholipid and triglyceride metabolism, enhanced brown fat thermogenesis, and promoted white fat browning. Additionally, in mouse models of obesity it increased energy expenditure and improved glucose handling while boosting leptin sensitivity through the inhibition of protein tyrosine phosphatase 1B [90]. However, long-term safety, optimal dosing regimens, and inter-individual variability in response remain insufficiently characterized.

Similarly, mangiferin, a phytochemical, was tested in a double-blind randomized controlled trial in patients with overweight and found to significantly lowered serum triglycerides and insulin resistance. These effects were associated with enhanced fatty acid and carbohydrate oxidation, increased β-hydroxybutyrate and acetoacetate levels, and higher lipoprotein lipase activity—demonstrating a mitochondrial-driven boost in energy expenditure and suppression of lipogenesis [91].

#### 3.5.5. Mitochondria-Targeted Antioxidants: Precision Approaches for Obesity

Targeting mitochondrial dysfunction directly is another promising avenue; recent studies in this area are summarized in Table 5. In one recent preclinical study, the inhibition of the small GTPase RalA, a key player in mitochondrial dynamics, prevented obesity-induced weight gain by enhancing fatty acid oxidation and preventing mitochondrial fragmentation [92].

Another noteworthy antioxidant is MitoTEMPO, a mitochondria-targeted compound. In murine models of obesity, MitoTEMPO reversed gut dysbiosis, reduced LPS-producing bacteria (e.g., *Bacteroidetes*), and restored *Bacillota* levels. This shift improved gut-derived SCFA levels and normalized metabolic pathways involving butyrate, propionate, and glutathione metabolism. Additionally, it mitigated cardiometabolic dysfunction, inflammation, and insulin resistance [93].

Likewise, Mitoquinone (MitoQ), a mitochondria-penetrating antioxidant, has shown beneficial effects on insulin sensitivity in humans. By accumulating within the inner mitochondrial membrane, MitoQ reduced oxidative stress under lipid overload conditions and promoted insulin-stimulated glucose uptake in skeletal muscle. These effects were linked to enhanced GLUT4 translocation without alterations to canonical insulin signalling pathways. Such findings support the potential use of MitoQ as an adjunctive strategy to counteract lipid-induced insulin resistance and metabolic disturbances associated with obesity [94].

Despite encouraging mechanistic findings, the clinical application of mitochondria-targeted antioxidants remains at an early stage. Key questions regarding optimal dosing, treatment duration, long-term safety, and potential off-target effects remain unresolved. Furthermore, the redox balance is tightly regulated, and excessive antioxidant supplementation may carry unintended consequences. Therefore, cautious interpretation is warranted until robust clinical data become available.

In summary, the microbiome–mitochondrial axis is emerging as a powerful and multifaceted therapeutic target in the management of obesity. This integrative perspective—encompassing diet, microbiota modulation, mitochondrial support, and targeted antioxidants—holds great promise for more sustainable and effective interventions in the fight against metabolic disorders.

In Table S1 in the Supplementary Material, all the studies mentioned throughout the review are detailed to reflect the therapeutic potential of innovative therapies targeting the microbiome–mitochondria axis.

### 3.6. An Integrative Model of the Microbiome–Mitochondria Axis in Obesity

Based on the evidence reviewed, we propose an integrative conceptual model of the microbiome–mitochondria axis in obesity. In this framework, diet-induced dysbiosis alters the production of microbial metabolites, including SCFAs, bile acids, and tryptophan-derived compounds, which modulate mitochondrial biogenesis, oxidative phosphorylation efficiency, and ROS production in metabolically active tissues such as adipose tissue, liver, and skeletal muscle.

In parallel, mitochondrial dysfunction characterized by impaired β-oxidation, altered dynamics (fusion/fission imbalance), and excessive ROS production may further compromise intestinal barrier integrity and immune regulation, thereby perpetuating dysbiosis and systemic low-grade inflammation.

**Figure 3:**
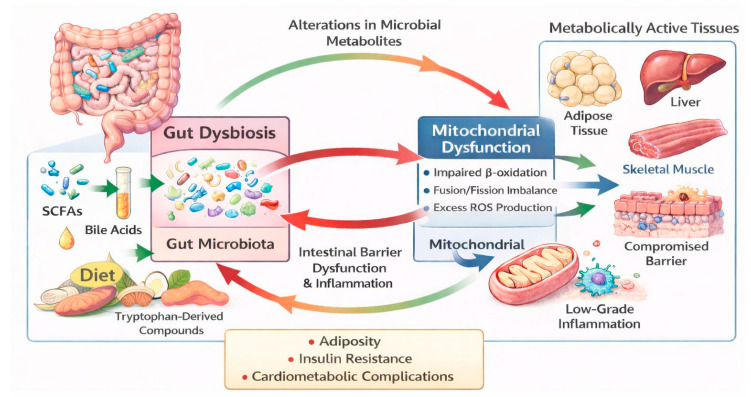
Microbiome–Mitochondria Axis in Obesity. Overview of the bidirectional relationship between gut microbiota and mitochondrial dysfunction in metabolic tissues. Diet-derived microbial metabolites (SCFAs, bile acids, and tryptophan-derived compounds) influence gut dysbiosis, intestinal barrier integrity, and inflammation, contributing to mitochondrial impairment and cardiometabolic complications.

This bidirectional loop may amplify metabolic disturbances and contribute to adiposity, insulin resistance, and cardiometabolic complications. Although supported mainly by mechanistic and preclinical evidence, this model provides a useful framework to guide future translational research (Figure 3).

#### Limitations

Despite the significance of the available evidence, several limitations must be acknowledged. First, there are still relatively few studies that comprehensively integrate the analysis of the microbiome–mitochondria axis as a potential therapeutic target in metabolic diseases. Much of the current knowledge is derived from indirect observations or studies that focused separately on either microbiome or mitochondrial functions, rather than examining their interaction as a unified mechanism.

In addition, the existing studies lack methodological standardization. Variations in study design, population characteristics, microbiome assessment techniques, and biomarkers of mitochondrial function hinder the comparison of results and the establishment of consistent conclusions. This heterogeneity limits the reproducibility and generalizability of the findings.

Furthermore, a significant proportion of the research has been conducted in animal models, making it difficult to extrapolate the results to human populations. Human clinical studies remain scarce and often involve small sample sizes or specific subgroups, further restricting the applicability of the conclusions.

Future research should therefore focus on the development of standardized methodologies and well-designed clinical trials to clarify how the microbiome–mitochondria axis can be effectively used as a therapeutic strategy.

## 4. Conclusions

Obesity is a chronic and multifactorial disease with a rapidly increasing global prevalence and a broad spectrum of associated comorbidities, underscoring the urgent need to optimize current therapeutic strategies. Although the pathophysiological mechanisms underlying obesity are not yet fully elucidated, accumulating evidence indicates that both mitochondrial dysfunction and gut dysbiosis contribute to its development and progression. Importantly, these alterations appear to interact in a bidirectional manner, forming a dynamic microbiome–mitochondria axis that may influence energy homeostasis, inflammatory signaling, and metabolic regulation.

The scoping methodology adopted in this review enabled a comprehensive mapping of current preclinical and clinical evidence, highlighting the growing recognition of microbiome–mitochondria crosstalk as a relevant component in obesity pathogenesis. While a substantial proportion of mechanistic insights derive from experimental models, emerging human studies support the biological plausibility of this axis as a therapeutic target.

From a translational perspective, the identification of reliable biomarkers capable of monitoring the microbiome–mitochondria axis remains a critical challenge. Potential candidates include circulating and fecal short-chain fatty acids, bile acid profiles, plasma lipopolysaccharide concentrations, markers of intestinal permeability (e.g., zonulin), and systemic inflammatory mediators. Complementarily, mitochondrial-related biomarkers—such as circulating mitochondrial DNA, PGC-1α expression, oxidative stress markers (e.g., malondialdehyde and 8-hydroxy-2′-deoxyguanosine), and assessments of mitochondrial respiration in peripheral blood mononuclear cells—may provide insight into systemic mitochondrial function. The integration of microbiome-derived metabolites with mitochondrial functional parameters could enable the development of composite biomarker panels with improved diagnostic and prognostic value.

Such multidimensional approaches may facilitate patient stratification and support the development of precision nutrition and personalized therapeutic strategies targeting the microbiome–mitochondria axis. In recent years, various interventions—including prebiotics, probiotics, postbiotics, natural bioactive compounds, and mitochondria-targeted antioxidants—have shown promising results in modulating this axis, particularly in preclinical models. Although further well-designed clinical trials are required to confirm their efficacy and long-term safety, these strategies represent an emerging and potentially impactful avenue in obesity research.

A deeper mechanistic understanding of microbiome–mitochondria interactions could not only contribute to improved obesity management but also enhance the prediction, prevention, and treatment of obesity-associated comorbidities. Continued integration of mechanistic, translational, and clinical research will be essential to fully elucidate the therapeutic potential of this complex and evolving biological axis.

## Figures and Tables

**Table 2:** Key findings on postbiotic interventions for obesity.

Outcome	Postbiotic	Model
↓ Dysbiosis	UDCA [84]	Human Murine
↓ Body weight		Murine
↑ Glucose tolerance		
↑ Energy expenditure		

**Table 3:** This table provides an overview of the main outcomes reported for various probiotic interventions in obesity models.

Outcome	Probiotic	Model
↓ Body weight or weight gain	*Bifidobacterium animalis* subsp. lactis A6 [75] *Bifidobacterium longum* [89]	Murine
↓ Fat mass	*Bifidobacterium animalis* subsp. lactis A6 [75]	
↓ LPS levels	*Bifidobacterium animalis* subsp. lactis A6 [75] *Limosillactobacillus reuteri* DSM 17938 [87]	
↓ Dysbiosis	*Bifidobacterium animalis* subsp. lactis A6 [75]	
↓ Food energy efficiency	*Akkermansia muciniphila* [86]	
↓ Obesity biomarkers		
↓ Inflammation	*Limosillactobacillus reuteri* DSM 17938 [87]	
↓ Insulin resistance		
↓ Mitochondrial dysfunction		
↓ Oxidative stress		
↓ Carbohydrate absorption	*Akkermansia muciniphila* [86]	
↑ Mitochondrial biogenesis or activity	*Bifidobacterium animalis* subsp. lactis A6 [75] Bifidobacterium *longum* [88]	
↑ Lipid metabolism	*Bifidobacterium longum* [88]	
↑ Energy loss	*Akkermansia muciniphila* [86]	
↑ Intestinal epithelial turnover		

**Table 4:** Documented metabolic, inflammatory and microbiota-related responses to natural bioactive compounds in murine and human obesity studies.

Outcome	Natural Compound	Model
↓ Body weight or weight gain	Rotundic acid [89]	Murine
↓ Dysbiosis		
↓ Adipose tissue mass	Rotundic acid [90]	
↓ Total body fat		
↓ Lipid levels	Mangiferin [91]	Individuals with obesity and hyperlipidemia (human)
↓ Insulin resistance		
↑ Brown fat thermogenesis	Rotundic acid [89]	Murine
↑ White fat browning	Rotundic acid [89]	
	Rotundic acid [90]	
↑ Energy expenditure	Mangiferin [91]	Individuals with obesity and hyperlipidemia (human)
↑ Oxidation of free fatty acids and carbohydrates		
↑ Lipid homeostasis	Rotundic acid [89]	Murine
↑ SCFAs		
↑ Glucose handling	Rotundic acid [90]	
↑ Leptin sensitivity		

**Table 5:** Overview of mitochondrial and metabolic responses to mitochondria-targeted antioxidant compounds in human and murine obesity models.

Outcome	Compound/Strategy	Model
↓ Body weight or weight gain	To inhibit the small GTPase RalA [92]	Murine
↓ Mitochondrial fragmentation		
↓ Cardiometabolic dysfunction	MitoTEMPO [93]	
↓ Inflammation		

↓ Insulin resistance	MitoQ [94]	Human
↓ Oxidative stress		
↓ Dysbiosis	MitoTEMPO [93]	Murine
↑ SCFAs		
↑ Fatty acid oxidation	To inhibit the small GTPase RalA [92]	
↑ Glucose uptake	MitoQ [94]	Human

## Data Availability

No new data were created or analyzed in this study.

## Supplementary Materials

The following supporting information can be downloaded at: https://www.mdpi.com/article/10.3390/medsci14010124/s1, Table S1: Summary of recent clinical and preclinical evidence supporting the therapeutic potential of the mitochondria–microbiome axis in obesity treatment, highlighting the limitations of conventional therapies and the advantages of alternative strategies targeting this novel pathway.

## Abbreviations

The following abbreviations are used in this manuscript:

ATP	Adenosine Triphosphate
Bas	Bile Acids
BCL-2	B-Cell Lymphoma 2
BMI	Body Mass Index
CAT	Catalase
Ce-RS3	Resistant Starch Type 3 from Canna Edulis
Gpx	Glutathione Peroxidase
LPS	Lipopolysaccharide
PGC-1α	Peroxisome Proliferator-Activated Receptor-Γ Coactivator-1α
PRISMA-P	Preferred Reporting Items for Systematic Reviews and Meta-Analyses Protocols
PRISMA-Scr	Preferred Reporting Items for Systematic Reviews and Meta-Analyses Extension for Scoping Reviews
ROS	Reactive Oxygen Species
RS5	Type 5 Resistant Starch
SBA	Secondary Bile Acids
SCFAs	Short-Chain Fatty Acids
SOD	Superoxide Dismutase
T2D	Type 2 Diabetes
UCP	Uncoupling Protein
UDCA	Ursodeoxycholate
