# Supplementary material for: Modulation of Microbiome–Mitochondria Axis as a Novel Approach for Treatment of Obesity: A Scoping Review
Source: Med Sci (Basel). 2026 Mar 6;14(1):124. doi: 10.3390/medsci14010124 (PMC13028557; doi:10.3390/medsci14010124)
Supplement: Supplementary file 1 [file medsci-14-00124-s001.zip › medsci-4149115-supplementary.pdf]

medical sciences

MDPI

Supplementary Materials: Modulation of Microbiome–Mitochondria Axis as a Novel Approach for Treatment of Obesity: A Scoping Review

Andreea Roxana Lista, Ciskey Vanessa Ayala Mosqueda, Rafael Palacios, María José García Mansilla, María Jesús Rodríguez Sojo, Ailec Ho Plágaro, Jorge Garcia Garcia, Julio Gálvez, Alba Rodríguez Nogales, and Antonio Jesús Ruiz Malagón, María José Rodríguez Sánchez

Table S1. Summary of recent clinical and preclinical evidence supporting the therapeutic potential of the mitochondria–microbiome axis in obesity treatment, highlighting the limitations of conventional therapies and the advantages of alternative strategies targeting this novel pathway.

<table>
<tr><td colspan="6">Conventional strategies:
• Modification of diet, physical exercise and lifestyle
• Adjuvant therapy (appetite suppressants, fat absorption inhibitors, GLP-1 analogs)</td></tr>
<tr><td>Limitations</td><td colspan="5">Difficulty in maintaining these strategies long-term often leads to regaining lost weight.</td></tr>
<tr><td colspan="6">Alternative strategies: microbiome-mitochondrial axis as a new therapeutic target</td></tr>
<tr><td rowspan="2"></td><td rowspan="2">Compound/ Strategy</td><td rowspan="2">Improvement of pathological aspects</td><td rowspan="2">Mechanism of action</td><td colspan="2">Recent research</td></tr>
<tr><td>Study design</td><td>Objective</td></tr>
<tr><td>Prebiotics</td><td>Inulin and rhubarb extract</td><td>To reduce weight gain and browning of brown adipose tissue
To enhance mitochondrial activity
To elevate levels of lipolytic markers
To stimulate the production of SCFA through</td><td>Microbiome modulation
To influence key transcription factors such as C/EBP and PPARγ, promoting an adaptive metabolic response</td><td>Preclinical study using a mouse model of metabolic disorders induced by a high-fat, high-sugar diet (n = 15 per group) [77].</td><td>To evaluate the impact of rhubarb and inulin-type fructans), individually and combined, on gut microbiota composition and metabolic outcomes in mice models of obesity</td></tr>
</table>

		rhubarb intervention			
	Ce-RS3	Favorable results proportional to the dose administered<br>To reverse dysbiosis<br>To decrease weight, inflammation, pathological biomarkers, and blood lipid levels	To promote the growth of beneficial bacteria such as *Bifidobacterium* and *Roseburia*	Preclinical studies in murine models of obesity (n = 8 per group) [78].	To assess the role of Ce-RS3 as an innovative approach to obesity management by analysing microbiome composition and metabolic profile
		To improve cholesterol levels and glutathione peroxidase activity<br>To reduce blood lipids<br>To inhibit fatty acid synthesis and elongation in mitochondria	To enhance populations of *Faecalibacterium*<br>To reduce *Ruminococcaceae* bacteria<br>To limit mitochondrial fatty acid synthesis	Randomised controlled trial in patients with mild hyperlipidaemia (n = 35-38 per group) [79].	To evaluate the effects of Ce-RS3 administration to patients with mild hyperlipidaemia, measuring serum lipid levels, microbiome alterations and antioxidant enzymes levels
	RS5	To decrease body weight<br>To enhance lipid metabolism	Regulation of gut microbiota, promotion of SCFA-producing bacteria *Bifidobacterium Turicibacter* and *Romboutsia.*, and improvement of redox balance particularly	Preclinical study using a diet-induced obesity rat model (n = 6 per group) [81].	To investigate the physiological and metabolic effects of RS5 in a rat model of diet-induced obesity

			via increased SOD levels		
		To increase microbial richness in the gut<br>To reduce body weight<br>To decrease blood and hepatic lipid levels and adipocyte size<br>To decrease glycemia	To improve lipid metabolism, reduce fat absorption, and regulate genes involved in lipogenesis and inflammation through SCFA activity	Preclinical study on a mouse model of hyperlipidaemia (n = 10 per group) [80].	To study the effects of RS5 supplementation on mice with hyperlipidaemia, observing changes in their microbiome, body weight, serum lipid levels and fat storage
	RT-90	To reduce weight and markers of inflammation and oxidative stress<br>Improvement in a diabetes biomarker	To reverse dysbiosis and promote a SCFA-producing microbiome<br><br>To increase activity of antioxidant enzymes such as GPx, SOD, and CAT	Preclinical study using mice as a model of obesity (n = 6) [82].	To determine the mechanism of obesity prevention by RT-90
Postbiotics	UDCA	To reduce body weight<br>To improve glucose tolerance	To positively modulate the gut microbiota<br>To enhance GLP-1 secretion in the ileum and upregulate UCP1 and PGC1α expression in brown adipose tissue, promoting energy expenditure	Translational study including:<br><br>Preclinical studies using murine models of obesity-prone and obesity-resistant mice (n = 5–8 per group)<br><br>Clinical analysis of adults with high BMI—classified as metabolically	To investigate how UDCA, a non-12-OH BA, modulates gut microbiota and metabolic pathways involved in obesity

				healthy (n = 121) or unhealthy (n = 62) [84].	
Probiotics	*Bifidobacterium animalis* subsp. lactis A6	To reduce body weight and fat mass, lower serum LPS levels, and modulate gut microbiota composition	To reduce the abundance of *Oscillibacter* and *Bilophila*, and to enhance the expression of key genes involved in mitochondrial activity, such as PGC-1α, ERRα, and UCP-1, in epididymal adipose tissue	Preclinical study in diet-induced obese mice (n = 6 for each group) [75].	To evaluate the potential of *Bifidobacterium animalis* subsp. lactis A6 to counteract obesity via mitochondrial and adipose tissue enhancement
	Akkermansia muciniphila	To alleviate diet-induced obesity<br>To reduce food energy efficiency<br>To increase energy expenditure<br>To promote energy loss via feces	To reduce the expression of perilipin2 in brown and white adipose tissue, a protein typically upregulated in obesity<br>To reduce carbohydrate absorption and increase intestinal epithelial turnover	*In vivo* study in a murine model of diet-induced obesity (n = 7 per group) [86].	To investigate whether pasteurized *Akkermansia muciniphila* reduces diet-induced obesity by modulating whole-body energy metabolism, including energy expenditure and physical activity
	Limosillactobacillus reuteri DSM 17938	To protect skeletal muscle and adipose tissue by preventing the onset of inflammation, insulin resistance and mitochondrial dysfunction<br>To exert beneficial effect on body composition, promoting protein mass accumulation while limiting	To reduce oxidative stress by preventing lipid peroxidation and limiting xanthine oxidase activity<br>To preserve mitochondrial antioxidant defense by maintaining SOD2 levels and avoiding excessive catalase activation	Preclinical studies using rats as a model of obesity (n = 8 for each group) [87].	To explore the potential of *Limosillactobacillus reuteri* DSM 17938 to protect skeletal muscle and adipose tissue from diet-induced inflammation and oxidative stress, emphasizing its effect on muscle mitochondria

https://doi.org/10.3390/medsci14010124

		fat mass expansion			
	Bifidobacterium longum	To attenuate weight gain<br>To potentially contribute to improved lipid metabolism and help reduce the risk of obesity	To increase AMPK phosphorylation in the liver and to suppress the expression of lipogenic genes<br>To upregulate genes involved in mitochondrial biogenesis and respiratory metabolism.	Preclinical experimental study using rats as an animal model of obesity (n = 4 per group) [88].	To evaluate the beneficial effects of milk fermented with different strains on energy metabolism and obesity prevention
Virome	Faecal virome transplantation	To reduce body weight<br>To improve glucose tolerance	To regulate microbiota composition<br>To modulate plasmic metabolome<br>To regulate obesity and T2D-related gene expression (such as the enhancement of Lepr, Ffar2, Igfbp2, Ppargc1a, and Klb, and the reduction of Socs3 and Myc)	Preclinical study in obese mice (n = 8 for each group) [42].	To evaluate the effect of transplanting faecal virome from lean to obese subjects, analyzing changes in gut microbiota and metabolic profile
Natural compounds	Rotundic acid	To improve weight management<br>To restore lipid homeostasis	To reverse dysbiosis by promoting the growth of SCFA-producing gut bacteria, such as *Alloprevotella*, *Bacteroides*, and *Desulfovibrio*, along with increasing the abundance of *Lactobacillus*	Preclinical study in hyperlipidemic rats (n = 7 per group) [89].	To assess the hypolipidemic potential of rotundic acid and elucidate its influence on gut microbiota and lipid metabolic pathways

			To regulate glycerophospholipid and triglyceride metabolism, carried out by the mitochondria<br>To enhance brown fat thermogenesis<br>To promote white fat browning		
		To increase energy expenditure<br>To improve glucose handling<br>To boost leptin sensitivity<br>Decrease in adipose tissue mass and total body fat percentage	Mechanisms mediated through the inhibition of negative regulators involved in leptin signaling pathways	Experimental investigation in mice with obesity induced (n = 5-8 for each group) [90].	To investigate the anti-obesity and anti-aging potential of rotundic acid by evaluating its role as a leptin sensitizer and its inhibitory effects on key negative regulators of leptin signaling
	Mangiferin	To normalize blood lipid levels and the insulin resistance index<br>Appears to improve energy expenditure	To promote the oxidation of free fatty acids and carbohydrates<br>To inhibit lipogenesis by acting on the mitochondria	Double-blind, randomized controlled trial conducted in patients with obesity and hyperlipidemia (n = 47-50 per group) [91].	To assess the impact of mangiferin on blood lipid profiles in a clinical trial involving overweight patients with hyperlipidemia
Mitochondria-Targeted Antioxidants	To inhibit the small GTPase RalA	To prevent obesity-induced weight gain<br>To enhance fatty acid oxidation and prevent mitochondrial fragmentation	To induce phosphorylation at Ser637 of Drp1, thereby attenuating its activity	Preclinical study in mouse models of obesity<br>(n = 5-13) [92].	Study of the effect of a high-fat diet on weight gain and mitochondrial fragmentation mediated by RalA, as well as the impact of its suppression on these processes

https://doi.org/10.3390/medsci14010124

	MitoTEMPO	To mitigate cardiometabolic dysfunction, inflammation, and insulin resistance	To reverse gut dysbiosis by reducing LPS-producing bacteria such as *Bacteroidetes* and restoring the abundance of *Firmicutes*<br>To improve SCFA levels and normalize metabolic pathways involving butyrate, propionate, and glutathione metabolism	Preclinical studies using murine models of obesity (n = 8 for each group) [93].	To assess the role of mitochondrial oxidative stress in diet-induced gut dysbiosis, and the contribution of gut microbiota to cardiometabolic disturbances in obese rats
	MitoQ	To improve insulin sensitivity in humans<br>To enhance glucose uptake in skeletal muscle under lipid overload	Accumulation in the inner mitochondrial membrane reduces oxidative stress and enhances GLUT4 translocation without affecting canonical insulin signaling	Clinical study in humans under controlled lipid overload conditions (n = 12), with complementary ex vivo analysis of skeletal muscle fibers [94].	To evaluate whether mitochondrial oxidative stress contributes to insulin resistance in human skeletal muscle under lipid overload

References

42. Rasmussen, T.S.; Mentzel, C.M.J.; Kot, W.; Castro-Mejía, J.L.; Zuffa, S.; Swann, J.R.; Hansen, L.H.; Vogensen, F.K.; Hansen, A.K.; Nielsen, D.S. Faecal virome transplantation decreases symptoms of type 2 diabetes and obesity in a murine model. *Gut* **2020**, *69*, 2122-2130, doi:10.1136/gutjnl-2019-320005.

75. Huo, Y.; Lu, X.; Wang, X.; Wang, X.; Chen, L.; Guo, H.; Zhang, M.; Li, Y. Bifidobacterium animalis subsp. lactis A6 Alleviates Obesity Associated with Promoting Mitochondrial Biogenesis and Function of Adipose Tissue in Mice. *Molecules (Basel, Switzerland)* **2020**, *25*, doi:10.3390/molecules25071490.

77. Régnier, M.; Van Hul, M.; Roumain, M.; Paquot, A.; de Wouters d'Oplinter, A.; Suriano, F.; Everard, A.; Delzenne, N.M.; Muccioli, G.G.; Cani, P.D. Inulin increases the beneficial effects of rhubarb supplementation on high-fat high-sugar diet-induced metabolic disorders in mice: impact on energy expenditure, brown adipose tissue activity, and microbiota. *Gut microbes* **2023**, *15*, 2178796, doi:10.1080/19490976.2023.2178796.

78. Wu, J.; Qiu, M.; Zhang, C.; Zhang, C.; Wang, N.; Zhao, F.; Lv, L.; Li, J.; Lyu-Bu, A.G.A.; Wang, T.; et al. Type 3 resistant starch from Canna edulis modulates obesity and obesity-related low-grade systemic inflammation in mice by regulating gut microbiota composition and metabolism. *Food & function* **2021**, *12*, 12098-12114, doi:10.1039/d1fo02208c.

79. Miao, T.; Zhang, X.; Zhang, C.; Wu, J.; Zhu, Y.; Xiao, M.; Zhang, N.; Zhong, Y.; Liu, Y.; Lin, Y.; et al. Type 3 resistant starch from Canna edulis reduce lipid levels in patients with mild hyperlipidemia through altering gut microbiome: A double- blind randomized controlled trial. *Pharmacological research* **2024**, *205*, 107232, doi:10.1016/j.phrs.2024.107232.
80. Li, H.; Wang, N.; Wu, J.; Tan, S.; Li, Y.; Zhang, N.; Yang, L.; Li, A.; Min, R.; Xiao, M.; et al. Characterization and Nutritional Intervention Effects of Canna edulis Type 5 Resistant Starch in Hyperlipidemia Mice. *Foods (Basel, Switzerland)* **2025**, *14*, doi:10.3390/foods14010092.
81. Zheng, B.; Wang, T.; Wang, H.; Chen, L.; Zhou, Z. Studies on nutritional intervention of rice starch-oleic acid complex (resistant starch type V) in rats fed by high-fat diet. *Carbohydrate polymers* **2020**, *246*, 116637, doi:10.1016/j.carbpol.2020.116637.
82. Liao, C.C.; Chen, S.Y.; Chen, Y.Y.; Huang, C.C.; Pan, R.Y.; Yen, G.C. Characterization of a novel type 4 resistant starch from tapioca and its obesity-preventive effects through gut microbiota modulation in high-fat diet-treated mice. *International journal of biological macromolecules* **2025**, *295*, 139577, doi:10.1016/j.ijbiomac.2025.139577.
84. Wei, M.; Huang, F.; Zhao, L.; Zhang, Y.; Yang, W.; Wang, S.; Li, M.; Han, X.; Ge, K.; Qu, C.; et al. A dysregulated bile acid-gut microbiota axis contributes to obesity susceptibility. *EBioMedicine* **2020**, *55*, 102766, doi:10.1016/j.ebiom.2020.102766.
86. Depommier, C.; Van Hul, M.; Everard, A.; Delzenne, N.M.; De Vos, W.M.; Cani, P.D. Pasteurized Akkermansia muciniphila increases whole-body energy expenditure and fecal energy excretion in diet-induced obese mice. *Gut microbes* **2020**, *11*, 1231-1245, doi:10.1080/19490976.2020.1737307.
87. Di Porzio, A.; Barrella, V.; Cigliano, L.; Mauriello, G.; Troise, A.D.; Scaloni, A.; Iossa, S.; Mazzoli, A. Diet-induced impairment of skeletal muscle and adipose tissue metabolic homeostasis and its prevention by probiotic administration. *Pflugers Archiv : European journal of physiology* **2025**, *477*, 223-239, doi:10.1007/s00424-024-03041-9.
88. Maruta, H.; Fujii, Y.; Toyokawa, N.; Nakamura, S.; Yamashita, H. Effects of Bifidobacterium-Fermented Milk on Obesity: Improved Lipid Metabolism through Suppression of Lipogenesis and Enhanced Muscle Metabolism. *International journal of molecular sciences* **2024**, *25*, doi:10.3390/ijms25189934.
89. Zeng, W.; Yang, B.; Wang, Y.; Sun, M.; Yang, W.; Cui, H.; Jin, J.; Zhao, Z. Rotundic acid alleviates hyperlipidemia in rats by regulating lipid metabolism and gut microbiota. *Phytotherapy research : PTR* **2023**, *37*, 5958-5973, doi:10.1002/ptr.8008.
90. Zhu, J.; An, Y.; Wang, X.; Huang, L.; Kong, W.; Gao, M.; Wang, J.; Sun, X.; Zhu, S.; Xie, Z. The natural product rotundic acid treats both aging and obesity by inhibiting PTP1B. *Life medicine* **2022**, *1*, 372-386, doi:10.1093/lifemedi/lnac044.
91. Na, L.; Zhang, Q.; Jiang, S.; Du, S.; Zhang, W.; Li, Y.; Sun, C.; Niu, Y. Mangiferin supplementation improves serum lipid profiles in overweight patients with hyperlipidemia: a double-blind randomized controlled trial. *Scientific reports* **2015**, *5*, 10344, doi:10.1038/srep10344.
92. Xia, W.; Veeragandham, P.; Cao, Y.; Xu, Y.; Rhyne, T.E.; Qian, J.; Hung, C.W.; Zhao, P.; Jones, Y.; Gao, H.; et al. Obesity causes mitochondrial fragmentation and dysfunction in white adipocytes due to RalA activation. *Nature metabolism* **2024**, *6*, 273-289, doi:10.1038/s42255-024-00978-0.
93. Ortega-Hernández, A.; Martínez-Martínez, E.; Gómez-Gordo, R.; López-Andrés, N.; Fernández-Celis, A.; Gutiérrez-Miranda, B.; Nieto, M.L.; Alarcón, T.; Alba, C.; Gómez-Garre, D.; et al. The Interaction between Mitochondrial Oxidative Stress and Gut Microbiota in the Cardiometabolic

Consequences in Diet-Induced Obese Rats. *Antioxidants (Basel, Switzerland)* **2020**, *9*, doi:10.3390/antiox9070640.

94. Fiorenza, M.; Onslev, J.; Henríquez-Olguín, C.; Persson, K.W.; Hesselager, S.A.; Jensen, T.E.; Wojtaszewski, J.F.P.; Hostrup, M.; Bangsbo, J. Reducing the mitochondrial oxidative burden alleviates lipid-induced muscle insulin resistance in humans. *Science advances* **2024**, *10*, eadq4461, doi:10.1126/sciadv.adq4461.

Disclaimer/Publisher's Note: The statements, opinions and data contained in all publications are solely those of the individual author(s) and contributor(s) and not of MDPI and/or the editor(s). MDPI and/or the editor(s) disclaim responsibility for any injury to people or property resulting from any ideas, methods, instructions or products referred to in the content.
